# Interoception Disorder and Insular Cortex Abnormalities in Schizophrenia: A New Perspective Between Psychoanalysis and Neuroscience

**DOI:** 10.3389/fpsyg.2021.628355

**Published:** 2021-07-01

**Authors:** Jessica Tran The, Pierre J. Magistretti, Francois Ansermet

**Affiliations:** ^1^Département d'études Psychanalytiques, Université de Paris, Paris, France; ^2^Agalma Foundation, Geneva, Switzerland; ^3^Faculté de Biologie et de Médecine, University of Lausanne, Lausanne, Switzerland; ^4^Brain Mind Institute, Federal Institute of Technology in Lausanne, Sion, Switzerland; ^5^Division of Biological and Environmental Sciences and Engineering, King Abdullah University of Science and Technology, Thuwal, Saudi Arabia; ^6^Department of Psychiatry, University of Geneva, Geneva, Switzerland

**Keywords:** interoception, insular abnormalities, schizophrenia, psychoanalysis, Freud, neuroscience, body perception, psychosis

## Abstract

The existence of disturbances in the perception of somatic states and in the representation of the body with the presence of cœnesthetic hallucinations, of delusional hypochondriac ideas or of dysmorphophobias is a recognized fact in the psychopathology of schizophrenia. Freudian psychoanalytic theory had accorded a privileged place to the alteration of the perception of the body in schizophrenia. Freud had attributed to these phenomena a primary and prodromal role in the psychopathology of psychosis. We propose to look at this theory in a new way, starting from the perspective of recent studies about the role of the insula in the perception and representation of somatic states, since this structure has been identified as underpinning the sense of interoception. The data in the neurobiological literature about abnormalities in the insular cortex in schizophrenia has shown that insula dysfunction could constitute one of the biological substrates of disorders of body perception in schizophrenia, and could be a source of the alteration of the sense of self that is characteristic of this psychiatric pathology. Moreover, this alteration could thus be involved in the positive symptomatology of schizophrenia.

## Introduction

The existence of disturbances in the perception of somatic states and in the representation of the body, together with the presence of cœnesthetic hallucinations and of delusional, hypochondriac ideas, or dysmorphophobias, is a recognized fact in the psychopathology of schizophrenia, which was observed in classical psychiatry. Freudian psychoanalytic theory had accorded an important place to this symptomology linked with the alteration of the perception of the body in schizophrenia. In particular, Freud had formulated the hypothesis that these phenomena would play an initial and prodromal role in the psychopathological process of psychosis, while the productive symptomology, and in particular delusional ideas and auditory hallucinations, would occur only in a second phase.

Recent discoveries in neurobiology, in particular A.D. Craig's work on interoception and the role of the posterior and anterior insula in the perception of somatic states and in the cortical representation of the image of the body (Craig, 2002), have opened up new avenues for research on the neurobiological substrates of the disorders in the perception of bodily states in schizophrenia. These results spurred researchers to investigate, using cerebral imaging techniques, the possibility of a structural and functional physiopathology in this cortical region in schizophrenic patients. The first studies have sought, in particular, to observe the existence of abnormalities in the anatomy of the insular cortex in schizophrenic patients, using data from structural magnetic resonance imaging (Parnas et al., 2005; Vollmer-Larsen et al., 2007; Jardri et al., 2008; Llorca et al., 2016). More recently, other studies have been concerned with functional abnormalities of the insula in these patients (Ardizzi et al., 2016). We propose, first of all, to review the Freudian theory of psychosis as a diachronic process with two steps, and Freud's theory of the existence of a hypochondriac “nucleus” of psychosis, in which cœnesthetic hallucinations and other alterations in the perception of somatic states play a major prodromal role. We will then return to the neurobiological data on the role of the insula in the perception of somatic states and in the representation of the image of the body. Next, we will review the results of various studies that have highlighted structural and functional abnormalities in the insular cortex in schizophrenic patients. We will study the possible correlations between the symptomatology tied to an alteration in the perception of somatic states in schizophrenia and the nature of the observed insular dysfunctions, in order to attempt to clarify how the physiopathology of the insula can appear to be one of the biological substrates of interoception disorder observable in the clinical pictures of schizophrenia. Finally, we will indicate new directions for research in the study of the correlations between the functional abnormalities of the insular cortex and the positive symptomatology of schizophrenia, with regard to the Freudian hypothesis of the primary character of the psychopathology linked with interoceptive perception in relation to delusional ideas and auditory hallucinations.

## The Freudian Hypothesis About the Existence of Somatic Perception Disorder in the First Step of Schizophrenia

Freud conceived psychosis as a temporal and diachronic psychopathological process, marked by the succession of “two steps” (Freud, 1924, p. 184). Even if auditory hallucinations and delusional ideas generally appear to be the most visible symptomatology in the clinical pictures of schizophrenia, this positive symptomatology belongs, according to Freud, only to the second step of the disease process. During the first step of illness, the patient experiences an “internal catastrophe” (Freud, 1911, p. 70), a collapse of his entire psychic world, and that it was only in a second step that delusion began to be active in the manner of a “an attempt at recovery”: “The delusional formation, which we take to be the pathological production, is in reality an attempt at recovery, a process of reconstruction” (Freud, 1911, p. 71).

Regarding the “first phase” of the psychopathological process at work in psychosis, Freud will theorize the existence of a hypochondriac “nucleus” (Freud, 1911, p. 37), which constitutes the stage that is the precursor to schizophrenia (Freud, 1916–1917): the beginning of the psychotic process is dominated by the weight of a cœnesthetic disorder, that is to say, a disturbance of the perception and the representation of the states of the body whose principal psychopathological manifestations are cœnesthetic hallucinations, dysmorphophobias, and hypochondriac ideas. Psychoanalytic theory thus hypothesizes the existence of a disturbance of the representation of somatic states and of the perception of the general state of the body, which characterizes the first phase of the disease process at work in psychosis.

Freud gave an illustration of this symptomatology of an alteration of body perception with the study of the clinical case of Schreber. Daniel Paul Schreber, president of the Dresden Court of Appeals, had been hospitalized in the Leipzig Clinic during different periods between 1884 and 1894, and recounted, in an autobiographical text, *Memoirs of My Nervous Illness*, his experience of psychosis (Schreber, 2000). Before the appearance of delusional ideas of persecution and of the first auditory hallucinations, the patient presents a serious disorder of the perception and the representation of his body, and the psychopathological symptomatology is dominated by the presence of hypochondriac ideas and by a great variety of cœnesthetic hallucinations, experienced as being extremely painful, and which bear witness to a massive alteration in the perception of the entirety of his bodily functioning. The patient explains that, during the first years of his illness, “he suffered destruction of individual organs of his body, of a kind which would have brought death to every other human being, that he lived for a long time without stomach, without intestines, bladder, almost without lungs, with smashed ribs, torn gullet, that he had at times eaten part of his own larynx…” (Schreber, 2000, p. 334). At the beginning of the illness, the clinical picture of Schreber is thus essentially dominated by a disorder of interoceptive perceptions, his body becoming the theater of an entire series of awful sensations, which have to do with his various organs, and which he describes as a permanent torture. Thereafter, the hallucinations and the hypochondriac ideas will evolve progressively into a delusion of persecution, since Schreber will attribute these sensations to the intervention of a supernatural exterior force:

“This was an extremely painful, caries-like state of the lower vertebrae” (Schreber, 2000, p. 151); “my lungs were for a long time the object of violent and very threatening attacks […]that for a time I seriously believed I had to fear a fatal outcome in consequence of pulmonary phthisis […] I had the definite feeling that my diaphragm was raised high in my chest to almost directly under my larynx and that there remained only a small remnant of lung in between…” (Schreber, 2000, p. 143).

This clinical case thus highlights the absolutely serious nature of the disturbances in the perception and the representation of somatic states that can be observed in the psychopathology of schizophrenia. If one juxtaposes Freudian ideas with the recent results of contemporary neuroscientists, this hypothesis can be translated in terms of interoception disorder. According to the definition introduced by Craig in 2002, interoception designates the “*sense of the physiological condition of the body*” (Craig, 2002). Various studies have attested to the frequency of cœnesthetic hallucinations and of the presence of unusual visceral and bodily sensations in schizophrenic patients (Parnas et al., 2005); this sensorial modality is particularly specific to schizophrenia, compared with different types of hallucinations found into other clinical pictures (Llorca et al., 2016). A study of the cerebral structures involved in the perception and the representation of bodily states can thus help to shed light on the biological substrates of this interoception disorder in schizophrenia.

## The Role of the Insular Cortex in Interoception

From an anatomical point of view, the structures involved in the perception and the representation of bodily states are essentially located in the somatosensorial cortex, the insula, and the parietal lobes, but also in the evolutionarily older structures such as the limbic system, the hypothalamus, and the brain stem (Damasio et al., 2013)—the right hemisphere being dominant for each of these regions. Several pathways are implicated in the neural processing of interoceptive signals, beginning with a rich interface between autonomic afferents and the central nervous system (Khalsa et al., 2018): primarily spinal, vagal, and glossopharyngeal afferents (Janig, 1996; Craig, 2002; Critchley and Harrison, 2013; Shivkumar et al., 2016), and several brainsterm: nucleus of the solitary tract, parabrachial nucleus, and periaqueductal gray, thalamus, hypothalamus, hippocampus, amygdala, and also cortical regions (insula and somatosensory cortices; Khalsa et al., 2009; Critchley and Harrison, 2013; Hassanpour et al., 2018). The representation of the current state of the organism is distributed, from a cerebral point of view, between a number of structures, both cortical and subcortical, and not located in a single anatomical spot. The information about the state of the viscera is thus projected in these different cerebral structures, or it is distributed in the form of neuronal “maps,” like the information coming from muscles and joints (Damasio, 2000a).

The works of the neurophysiologist Arthur Craig in particular made it possible to indicate the crucial role of the insula in the representation of internal states of the body, and to describe the neuronal foundations of the *interoceptive* sense. The insula is shown to be a crucial cortical region for the perception of somatic states, a source of this “sense of the physiological condition of the body” (Craig, 2002). The data from functional imaging has also contributed to highlighting the crucial role of the insula in the treatment of emotional stimuli. The work of Craig (2009), Damasio (2003), and more recently of Critchley et al. (2004), has been able to confirm James's intuitions, according to which the emotions rely on the perception of the state of the different parts of the organism (James, 1890).

It has been established that the human insular cortices are involved in processing somatic states and body feelings (Damasio et al., 2000; Kupers et al., 2000; Brooks et al., 2002; Craig, 2002, 2010). But the Craig's view from which insula is the only source of feeling states perception in humans (Craig, 2009, 2011) is quite controversial, and L. F. Barett explicitly rejects it (Barrett and Simmons, 2015). Damasio et al. (2013) have studied a patient whose insular cortices were destroyed bilaterally as a result of Herpes simplex encephalitis, but whose all aspects of feelings were intact. From this study, they established that eventually the signals used to assemble the neural substrates of feelings hail from different sectors of the body and are conveyed by neural and humoral pathways to complex and topographically organized nuclei of the brain stem, prior to being conveyed again to cerebral cortices in the somatosensory, insular, and cingulate regions. They suggest that the neural substrate of feeling states is to be found first subcortically and then secondarily repeated at cortical level: the subcortical level would ensure basic feeling states while the cortical level would largely relate feeling states to cognitive processes such as decision-making and imagination.

However, insular cortices are an important cerebral region for the interoception. As Wyliea and Tregellas's article emphasized, interoception in the insula is demonstrated by its response during the perception of changes in the physiological state of the body (Wyliea and Tregellas, 2010), such as the observation of heart palpitations (Critchley et al., 2004), biofeedback using the changes of skin conductance (Critchley et al., 2002), thermal pain (Kong et al., 2006), pain induced electrically (Singer et al., 2004), and light touch (Lovero et al., 2009). In general, the insula is activated in the processing of signals tied to a series of somatic states: pain, body temperature, itching, tickling, visceral sensation, the state of smooth muscles, and of blood vessels. The data from functional neuroimaging has also shown an activation of the right anterior insular and orbitofrontal cortices during sexual excitation, and even during the onset of positive or negative sensations engendered by listening to a musical stimulus (Craig, 2002). This cerebral structure would thus make it possible to generate a direct thalamocortical representation of the state of the body in primates on the basis of the processing of all of these signals that convey information on somatic states.

The insula is thus at work in the processing of numerous interoceptive stimuli. But the data furnished by neuroimaging has also spurred authors such as Craig and Damasio to claim that the perception of physiological changes in the body and their evolution over time is at the origin of the sense of self (Damasio, 2000b; Craig, 2009). Furthermore, the interoceptive consciousness of the body as a whole also makes possible a representation of our organism as an entity distinct from the exterior environment: the insula thus appears to be a cortical region that is decisive for the distinction between the self and the “non-self” (Kircher et al., 2001; Devue et al., 2007).

## Structural Abnormalities of the Insular Cortex in Schizophrenia

The frequency of cœnesthetic hallucinations and of unusual corporeal and visceral sensations (such as “migrating inner sensations wandering through the body, electric, or thermal feelings, abnormal sense of pulling/pressure or heaviness/emptiness inside of the body, and dysesthetic crises involving the vegetative system” (Ardizzi et al., 2016), attested to by several neuroscientific studies (Parnas et al., 2005; Vollmer-Larsen et al., 2007; Jardri et al., 2008; Llorca et al., 2016)—suggests the existence of a grave alteration in the sensitivity of patients to internal bodily signals, just like what Freud had described in his observations on the Schreber case. This symptomatology relating to an alteration in interoception in schizophrenia could be put into perspective with the data attesting to the existence of structural and functional abnormalities of the insular cortex in schizophrenic patients. If one brings these different elements together, the physiopathology of the insula may appear to be one of the neurobiological substrates of disorders of perception and of the representation of somatic states observable in the clinical pictures of schizophrenia. The first neuroimaging studies aiming to explore the role of the insula in schizophrenia sought, in particular, to observe the existence of abnormalities in the anatomy of the insular cortex in schizophrenic patients, using structural magnetic resonance imaging. One of the first studies, conducted in 2000 by American researchers at the University of Iowa, having explored the morphology of the insular cortex in these patients, observed a reduction in the cortical surface and in the volume of left insular gray matter—a reduction amplified according to the severity of the patients' symptomatology: the more severe the symptomatology presented by the patients, the more one observes a significant diminution of the volume and the surface of the insular cortex (Jang et al., 2006). But other studies have subsequently shown the existence of a modification of the morphology of the frontal temporal sides of the right insula (which, as we have seen, is the region involved in the perception of interoceptive signals) in schizophrenic patients compared with healthy patients, without finding abnormalities in the left insular cortex (Wyliea and Tregellas, 2010). In addition to a simple reduction in the volume of the insular cortical surface, D. P. Jang and his collaborators showed that the morphological deformations concerning the length of the right side of the insula is smaller among patients than among control subjects. They also underscored that this deformation could have appeared at a relatively early stage of development of the patients, and they hypothesized a neurodevelopmental origin of this structural difference in the form of the surface of the insula (a deformation that could also be associated with frontal-temporal abnormalities; Jang et al., 2006).

The diminution of the insular gray matter, both bilateral and progressive over the course of the chronic development of the pathology, appears to be a recurring result in various studies, but it is not currently clear if these deficits are localized in a particular sub-region of the insula (Wyliea and Tregellas, 2010), since they have been observed in the anterior (Makris et al., 2006) as well as posterior insula (Saze et al., 2007), or in both regions (Kasai et al., 2003; Takahashi et al., 2005). The same is true for the observation of the thickness of the insular cortical layer—diminutions on the left side, on the right side, or on both sides have been reported (Wyliea and Tregellas, 2010). Post-mortem studies have also shown a diminution in the number of neurons in the outer layers of the insular cortex, as well as a reduction in neuronal and glial cells in the second layer (Pennington et al., 2008a). Moreover, an abnormal protein expression in this layer of the insular cortex has also been observed in schizophrenic patients—as abnormally expressed proteins are involved in neuronal growth, morphogenesis, and synaptic connections, they thus affect different levels of neuroplasticity (Pennington et al., 2008b).

Nevertheless, as Haukvik emphasized in a general review, published in 2013, on the studies dealing with structural brain imaging in schizophrenic patients, even if these patients tend to have a thinner cortex in certain parts of the brain such as the insula, enlarged lateral ventricles, and smaller volumes of hippocampus, this data still does not suffice in order to distinguish, using structural brain imaging, schizophrenic individuals from those who do not present any psychiatric disorder (Haukvik et al., 2013). There is thus currently no “objective” and measurable criterium correlated in a reliable way with a diagnosis of schizophrenia, and this disease can be diagnosed today only in an exclusively clinical manner.

## The Role of Insula Dysfunction in the Disturbance of Interoception in Schizophrenia

The development of the recourse to functional MRI techniques in psychiatry for the purposes of research has also helped to indicate the potential role of different cerebral regions in the physiopathology of schizophrenia. Even if no anatomical region of the brain has thus far been judged to be essential, functional imaging has contributed in particular to a better understanding of the relations between these zones and the functional networks that connect them. Neuroscientific studies on schizophrenia have initially focused, basically, on the hippocampus and the prefrontal cortex (Sigurdsson and Duvarci, 2016), but the existence of insula dysfunction has also begun to be explored more recently.

Many neuroscientific studies linked self-consciousness to the processing and integration of multisensory bodily signals, but in particular, certain elements from functional imaging data contribute to the hypothesis of a link between the disturbance of perception and of the representation of the states of the body in schizophrenia, and the possibility of insula dysfunction.

Heydrich and Blanke (2013) performed quantitative lesion analysis in a group of patients with heautoscopy hallucination and compared the location of brain damage with those of control patients suffering from complex visual hallucinations. During heautoscopy, subjects report strong self-identification with the second own body, often associated with the experience of existing at and perceiving the world from two places at the same time. Heydrich and Blanke found that heautoscopy was associated with lesions to the left posterior insula.

In another functional magnetic resonance imaging study, Ebisch et al. (2014) provide new evidence for a cortical link between aberrant self-experience and social cognition in first-episode schizophrenia. Ventral premotor cortex and posterior insula are candidate brain regions underlying disturbances in both self-experience and self-other relationship due to their processing of predominantly externally guided and internally guided like interoception. Their results show aberrant functional interactions of right Ventral premotor cortex and posterior insular cortex with posterior cingulate cortex, a midline region that has been shown central in mediating self-experience. Their results show more particularly increased aberrant functional interactions are positively correlated with basic symptoms like subjective self-experience disturbances.

Otherwise, the question of the processing of pain, which significantly involves the insula from a neurobiological point of view, appears also to be a major issue for the problematic of the perception of the states of the body in psychosis. A high involvement of the posterior and anterior insula in the perception of pain has been observed. Moreover, this region is activated not only in cases of pain relating to real organic lesions, but also in cases of patients suffering from chronic and neuropathic pain. The posterior insula receives, in particular, sensorial input from the somatosensorial cortices and from the ventroposterior laterals of the thalamus, this sensorial information being integrated in the anterior insular to produce, through the connections with the limbic system and the prefrontal cortex, a subjective evaluation of the emotional elements of pain (Haukvik et al., 2013). The functions of these two sub-regions are thus complementary: the posterior insula codes, in a bilateral manner, the experience of thermal pain, while the anterior bilateral insula participates in the evaluation of the intensity of pain. The two regions are activated when a subject feels, subjectively, a sensation of pain (Kong et al., 2006).

Moreover, the vision of a person who seems to be the victim of sharp pains, who produces a feeling of “empathic pain,” activates solely the anterior insula (Oschner et al., 2008). On the contrary, the experiments aiming to distract a subject suffering from pain—for example by making him laugh or inciting him to concentrate on something else—imply a diminution of the activation of the anterior insula, observed by functional imaging (Brooks et al., 2002). Thus, the subjective evaluation of pain seems to be processed in the anterior insula in a somewhat autonomous and independent manner with regard to the state of the body as it is perceived at the level of the posterior insula. There would thus be a discontinuity between the reality of the physiological state of the organism, and the perception and representation of this state that is empirically observable and corroborated from a neurobiological point of view. Damasio describes this phenomenon as “either a body loop or an ‘as if' loop,” that is to say, as virtual neuronal maps of the body, which do not correspond to the reality of the state of the organism at a given moment (Damasio, 1996). This simulation mechanism thus operates exclusively within the central nervous system, by “short-circuiting” the body, rather than there being an authentic perception of a bodily modification happening in reality in the organism at a given moment.

Cœnesthetic hallucinations consist in a subjective sensation of pain, which does not correspond to the reality of the physiological state of the body, since this sensation has no anatomical mooring. Nevertheless, no functional imaging study has so far attempted to evaluate precisely what could be the possible role of the insula in the processing of pain in schizophrenia, and in particular its involvement in the cases of cœnesthetic hallucinations generally perceived by the patient as disagreeable or painful. It is pertinent to explore if insular cortex abnormalities in schizophrenia could be correlated with the presence of cœnesthetic hallucinations and hypochondriac delusional ideas. More generally, alteration of the interoceptive precision in schizophrenia was observed (Ardizzi et al., 2016). But it would also be expedient to determine if significant correlations can be observed between the alteration of interoception in schizophrenic patients and anatomic-functional abnormalities of the insular cortex. Finally, if a correlation between the severity of the interoception disorder and the clarity of a positive symptomatology in schizophrenic patients has been observed, it would likewise bear studying these links more closely in order to observe whether the interoception disorder in schizophrenia could, secondarily, engender abnormalities in higher cognitive functions, and if it could be at the origin, as Freud postulated, of auditory hallucinations and delusional ideas.

Indeed, Craig and Damasio have defended the thesis that the perception and the representation of states of the body plays a major role in the advent of a sense of self (Damasio, 2000b; Craig, 2009). It can thus be hypothesized that the anatomical-functional abnormalities of the insular cortex, in so far as they can be considered to alter the first level of the sense of self, which Damasio has described as the “proto-self” (Damasio, 2000b) with regard to the perception and representation of states of the body, could eventually engender disorders for the higher forms of feeling that constitute the sense of self. Thus, if one follows the psychoanalytic hypothesis of a prodromal and nodal role of a disorder of perception and of the representation of the state of the body in psychosis, it would be expedient to explore the repercussions of this disorder for the more complex forms of the sense of self (and in particular for the “core self and autobiographical self”; Damasio, 2000b) in order to find out whether the alterations of higher levels of consciousness in psychosis, involved in delusional ideas and auditory hallucinations (which affect exteroceptive perception and, more generally, the relation to the surrounding environment) could be conceived of as continuous with the alterations of the “proto-self,” which relate to the disturbance of perception and of the representation of the states of the body. It could thus be supposed that the disorder of interoception may have consequences for more complex cognitive functions, and that it may be at the origin of the productive symptomatology, which would thus need to be considered to be chronologically secondary.

## Embodied Predictive Interoception Coding Model and Insular Abnormalities

Intuition suggests that perception follows sensation and therefore bodily feelings originate in the body. However, recent evidence goes against this logic: interoceptive experience may largely reflect limbic predictions about the expected state of the body that are constrained by ascending visceral sensations. In this Opinion article, we introduce the Embodied Predictive Interoception Coding model, which integrates an anatomical model of corticocortical connections with Bayesian active inference principles, to propose that agranular visceromotor cortices contribute to interoception by issuing interoceptive predictions. We then discuss how disruptions in interoceptive predictions could function as a common vulnerability for mental and physical illness.

The hypothesis that insular abnormalities in schizophrenia can be associated with schizophrenia disturbance of perception of the states of the body and more generally with schizophrenia as a pathology of self, may be completed with contributions of the embodied predictive interoception coding model. According to Friston (2010) and Clark (2013) views, Lisa Feldman Barrett and W. Kyle Simmons suggest that bodily feelings from the body are not first sensations and become after perceptions, but that interoceptive experience may largely reflect limbic predictions about the expected state of the body that are constrained by ascending visceral sensations.

We observed that Craig (2009) had asserted that the insular cortex plays a part in transforming ascending interoceptive signals into a “global emotional moment,” with the anterior insula being a crucial area for human awareness. But Barrett and Simmons (2015) criticized his views, and they proposed a model that does not focus on the anterior insula as a necessary area for consciousness or emotional awareness *per se*. This is because, in their model, multiple pathways within the combined cortical interoceptive network and the ascending pathways can construct interoceptive perceptions thereby making conscious presence and emotional awareness more resilient to brain injury.

They have introduced the Embodied Predictive Interoception Coding model, which integrates an anatomical model of corticocortical connections with Bayesian active inference principles, to propose that agranular visceromotor cortices and insula contribute to interoception by issuing interoceptive predictions. According to the EPIC model, Interoceptive sensation is largely prediction: “there is an interoceptive system in the brain in which agranular cortices send visceromotor predictions to the body and transmit interoceptive predictions about the viscerosensory consequences of those predictions” (Barrett and Simmons, 2015). Barrett and Simmons suggest that agranular visceromotor cortices (including the cingulate cortex, the posterior ventral medial prefrontal cortex, the posterior orbitofrontal cortex, and the most ventral portions of the anterior insula) estimate the balance between the autonomic, metabolic, and immunological resources that are available to the body and the predicted requirements of the body, based on past experience. They also explain that disruptions in interoceptive predictions could function as a common vulnerability for mental and physical illness. The EPIC model predicts that aberrant interoceptive predictions can lead to mental diseases, for example depression (Barrett et al., 2016). Philip Gerrans has also use this model for understanding the phenomenon of pain asymbolia, a condition in which nociceptive signals of bodily damage are not attributed to the self (Gerrans, 2020). According to his view, we can suggest that insular abnormalities showed by fMRI in schizophrenia may also generate interoception predictive disorders, and have an impact in body states perception and representation, and also in self-awareness.

Furthermore, alterations of the insular cortex functioning and interoceptive system were described in many different clinical contexts, neurological and psychiatric, such as depression and anxiety disorders (Vicario et al., 2020). Nevertheless, the presence of insular alterations in other neuropsychiatric disorders does not straight determine cœnesthetic hallucinations or delusional ideas and auditory hallucinations. A future direction of research is to investigate the reasons why these insular abnormalities manifest differently in schizophrenia and other mental disorders.

## Conclusion

The demonstration of the critical role of the insula in the perception of states of the body and in the generation of a cortical representation of somatic states opens a promising field of research in psychiatry, and makes it possible to pursue new avenues of investigation on the physiopathology of the insula in schizophrenia. Some studies have indeed shown the existence of significant anatomical and functional abnormalities of the cortex of the insula in schizophrenic patients, which could constitute the biological substrate of the alteration of interoception observed in these patients. These results help to shed new light on the importance accorded by Freudian psychoanalytic theory to the symptoms connected to corporeal perception, such as cœnesthetic hallucinations and hypochondriac ideas.

If the insula is particularly involved in the processing of pain, it would be useful to explore in greater detail what might be the role played by insula dysfunction in the disturbances of the subjective processing of pain in schizophrenic patients, in order to better understand the involvement of abnormalities of the cortical structure in cœnesthetic hallucinations, generally perceived by patients as disagreeable and painful. In particular, it would be worthwhile to study whether or not the substance of these abnormalities is correlated with the presence of cœnesthetic hallucinations in schizophrenia.

Finally, if a correlation between the severity of the interoception disorder and the clarity of a positive symptomatology in schizophrenic subjects has been observed, these links should be studied in greater detail in order to discern if the interoception disorder in schizophrenia, insofar as it engenders an alteration in the sense of self, could have, as a secondary consequence, abnormalities in higher cognitive functions, and be a source of auditory hallucinations and delusional ideas.

## Author Contributions

JT is the main contributor of this paper as part of her Ph.D. thesis. FA and PM as supervisors, contributed to the conception and development of the research, and they revised critically the manuscript for intellectual content. All authors contributed to the article and approved the submitted version.

## Conflict of Interest

The authors declare that the research was conducted in the absence of any commercial or financial relationships that could be construed as a potential conflict of interest.

## References

[B1] Ardizzi M. Ambrosecchia M. Buratta L. Ferri F. Peciccia M. Donnari S.. (2016). Interoception and positive symptoms in schizophrenia. Front. Hum. Neurosci. 10:379. 10.3389/fnhum.2016.00379 27512369 PMC4961721

[B2] Barrett L. F. Quigley K. S. Hamilton P. (2016). An active inference theory of allostasis and interoception in depression. Philos. Trans. R. Soc. Lond. B Biol. Sci. 371:20160011. 10.1098/rstb.2016.0011 28080969 PMC5062100

[B3] Barrett L. F. Simmons W. K. (2015). Interoceptive predictions in the brain. Nat. Rev. Neurosci. 16, 419–429. 10.1038/nrn3950 26016744 PMC4731102

[B4] Brooks J. C. W. Nurmikko T. J. Bimson W. E. Singh K. D. Roberts N. (2002). fMRI of thermal pain: effects of stimulus laterality and attention. Neuroimage 15, 293–301. 10.1006/nimg.2001.0974 11798266

[B5] Clark A. (2013). Whatever next? Predictive brains, situated agents, and the future of cognitive science. Behav. Brain Sci. 36, 181–204. 10.1017/S0140525X12000477 23663408

[B6] Craig A. D. (2002). How do you feel? Interoception: the sense of the physiological condition of the body. Nat. Rev. Neurosci. 3, 655–666. 10.1038/nrn894 12154366

[B7] Craig A. D. (2009). How do you feel-now? The anterior insula and human awareness. Nat. Rev. Neurosci. 10, 59–70. 10.1038/nrn2555 19096369

[B8] Craig A. D. (2010). The sentient self. Brain Struct Funct. 214, 563–577. 10.1007/s00429-010-0248-y 20512381

[B9] Craig A. D. (2011). Significance of the insula for the evolution of human awareness of feelings from the body. Ann. N. Y. Acad. Sci. 1225, 72–82. 10.1111/j.1749-6632.2011.05990.x 21534994

[B10] Critchley H. D. Harrison N. A. (2013). Visceral influences on brain and behavior. Neuron 77, 624–638. 10.1016/j.neuron.2013.02.008 23439117

[B11] Critchley H. D. Melmed R. N. Featherstone E. Mathias C. J. Dolan R. J. (2002). Volitional control of autonomic arousal: a functional magnetic resonance study. Neuroimage 16, 909–919. 10.1006/nimg.2002.1147 12202079

[B12] Critchley H. D. Wiens S. Rotshtein P. Ohman A. Dolan R. J. (2004). Neural systems supporting interoceptive awareness. Nat. Neurosci. 7, 189–195. 10.1038/nn1176 14730305

[B13] Damasio A. Damasio H. Tranel D. (2013). Persistence of feelings and sentience after bilateral damage of the insula. Cereb. Cortex 23, 833–846. 10.1093/cercor/bhs077 22473895 PMC3657385

[B14] Damasio A. R. (1996). The somatic marker hypothesis and the possible functions of the prefrontal cortex. Philos. Trans. R. Soc. Lond. B Biol. Sci. 351, 1413–1420. 10.1098/rstb.1996.0125 8941953

[B15] Damasio A. R. (2000a). Descart's Error: Emotion, Reason, and the Human Brain. Fort Mill, SC: Quill.

[B16] Damasio A. R. (2000b). The Feeling of What Happens: Body, Emotion in the Making of Consciousness. Boston, MA: Mariner Books.

[B17] Damasio A. R. (2003). Mental self: the person within. Nature 423:227. 10.1038/423227a 12748620

[B18] Damasio A. R. Grabowski T. J. Bechara A. Damasio H. Ponto L. L. B. Parvizi J.. (2000). Subcortical and cortical brain activity during the feeling of self-generated emotions. Nat. Neurosci. 3, 1049–1056. 10.1038/79871 11017179

[B19] Devue C. Collette F. Balteau E. Degueldre C. Luxen A. Maquet P.. (2007). Here I am: the cortical correlates of visual self-recognition. Brain Res. 1143, 169–182. 10.1016/j.brainres.2007.01.055 17306235

[B20] Ebisch S. J. Mantini D. Northoff G. Salone A. De Berardis D. Ferri F.. (2014). Altered brain long-range functional interactions underlying the link between aberrant self-experience and self-other relationship in first-episode schizophrenia. Schizophr. Bull. 40, 1072–1082. 10.1093/schbul/sbt153 24191160 PMC4133668

[B21] Freud S. (1911). Psycho-Analytic Notes on an Autobiographical Account of a Case of Paranoia. S.E. 12. London: Hogarth. p. 3–82.

[B22] Freud S. (1916–1917). “The common neurotic state,” in Introductory Lectures on Psycho-Analysis 1916-1917. S.E. 16. (London: Hogarth), 378–391.

[B23] Freud S. (1924). The Loss of Reality in Neurosis and Psychosis. S.E. 19. London: Hogarth. p. 183–190.

[B24] Friston K. (2010). The free-energy principle: a unified brain theory? Nat. Rev. Neurosci. 11, 127–138. 10.1038/nrn2787 20068583

[B25] Gerrans P. (2020). Pain asymbolia as depersonalization for pain experience. An interoceptive active inference account. Front. Psychol. 11:523710. 10.3389/fpsyg.2020.523710 33192765 PMC7658103

[B26] Hassanpour M. S. Simmons W. K. Feinstein J. S. Luo Q. Lapidus R. Bodurka J.. (2018). The insular cortex dynamically maps changes in cardiorespiratory interoception. Neuropsychopharmacology 43, 426–434. 10.1038/npp.2017.154 28726799 PMC5729563

[B27] Haukvik U. K. Hartberg C. B. Agartz I. (2013). Schizophrenia - what does structural MRI show? Tidsskr. Nor. Laegeforen. 133, 850–853. 10.4045/tidsskr.12.1084 23612107

[B28] Heydrich L. Blanke O. (2013). Distinct illusory own-body perceptions caused by damage to posterior insula and extrastriate cortex. Brain 136(Pt 3), 790–803. 10.1093/brain/aws364 23423672

[B29] James W. (1890). The Principles of Psychology. Cambridge, MA: Harvard University Press.

[B30] Jang D. P. Kim J. J. Chung T. S. An S. K. Jung Y. C. Lee J. K.. (2006). Shape deformation of the insula in schizophrenia. NeuroImage 32, 220–227. 10.1016/j.neuroimage.2006.01.032 16730197

[B31] Janig W. (1996). Neurobiology of visceral afferent neurons: neuroanatomy, functions, organ regulations and sensations. Biol. Psychol. 42, 29–51. 10.1016/0301-0511(95)05145-7 8770369

[B32] Jardri R. Pins D. Thomas P. (2008). A case of fMRI-guided rTMS treatment of coenesthetic hallucinations. Am. J. Psychiatry 165, 1490–1491. 10.1176/appi.ajp.2008.08040504 18981081

[B33] Kasai K. Shenton M. E. Salisbury D. F. Onitsuka T. Toner S. K. Yurgelun-Todd D.. (2003). Differences and similarities in insular and temporal pole MRI gray matter volume abnormalities in first-episode schizophrenia and affective psychosis. Arch. Gen. Psychiatry. 60, 1069–1077. 10.1001/archpsyc.60.11.1069 14609882

[B34] Khalsa S. S. Adolphs R. Cameron O. G. Critchley H. D. Davenport P. W. Feinstein J. S.. (2018). Interoception and mental health: a roadmap. Biol. Psychiatry Cogn. Neurosci. Neuroimaging. 3, 501–513. 10.1016/j.bpsc.2017.12.004 29884281 PMC6054486

[B35] Khalsa S. S. Rudrauf D. Feinstein J. S. Tranel D. (2009). The pathways of interoceptive awareness. Nat. Neurosci. 12, 1494–1496. 10.1038/nn.2411 19881506 PMC2787640

[B36] Kircher T. T. Senior C. Phillips M. L. Rabe-Hesketh S. Benson P. J. Bullmore E. T.. (2001). Recognizing one's own face. Cognition 78, B1–B15. 10.1016/S0010-0277(00)00104-9 11062324

[B37] Kong J. White N. S. Kwong K. K. Vangel M. G. Rosman I. S. Gracely R. H.. (2006). Using fMRI to dissociate sensory encoding from cognitive evaluation of heat pain intensity. Hum. Brain Mapp. 27, 715–772. 10.1002/hbm.20213 16342273 PMC6871429

[B38] Kupers R. C. Gybels J. M. Gjedde A. (2000). Positron emission tomography study of a chronic pain patient successfully treated with somatosensory thalamic stimulation. Pain 87, 295–302. 10.1016/S0304-3959(00)00295-5 10963909

[B39] Llorca P. M. Pereira B. Jardri R. Chereau-Boudet I. Brousse G. Misdrahi D.. (2016). Hallucinations in schizophrenia and Parkinson's disease: an analysis of sensory modalities involved and the repercussion on patients. Sci. Rep. 6:38152. 10.1038/srep38152 27905557 PMC5131286

[B40] Lovero K. L. Simmons A. N. Aron J. L. Paulus M. P. (2009). Anterior insular cortex anticipates impending stimulus significance. Neuroimage 45, 976–983. 10.1016/j.neuroimage.2008.12.070 19280711 PMC3758834

[B41] Makris N. Goldstein J. M. Kennedy D. Hodge S. M. Caviness V. S. Faraone S. V.. (2006). Decreased volume of left and total anterior insular lobule in schizophrenia. Schizophr. Res. 83, 155–171. 10.1016/j.schres.2005.11.020 16448806

[B42] Oschner K. N. Zaki J. Hanelin J. Ludlow D. H. Knierim K. Ramachandran T.. (2008). Your pain or mine? Common and distinct neural systems supporting the perception of pain in self and other. Soc. Cogn. Affect. Neurosci. 3, 144–160. 10.1093/scan/nsn006 19015105 PMC2555461

[B43] Parnas J. Møller P. Kircher T. Thalbitzer J. Jansson L. Handest P. (2005). EASE: examination of anomalous self-experience. Psychopathology 38, 236–258. 10.1159/000088441 16179811

[B44] Pennington K. Dicker P. Dunn M. J. Cotter D. R. (2008a). Proteomic analysis reveals protein changes within layer 2 of the insular cortex in schizophrenia. Proteomics 8, 5097–5107. 10.1002/pmic.200800415 19003868

[B45] Pennington K. Dicker P. Hudson L. Cotter D. R. (2008b). Evidence for reduced neuronal somal size within the insular cortex in schizophrenia, but not in affective disorders. Schizophr. Res. 106, 164–171. 10.1016/j.schres.2008.08.022 18805671

[B46] Saze T. Hirao K. Namiki C. Fukuyama H. Hayashi T. Murai T. (2007). Insular volume reduction in schizophrenia. Eur. Arch. Psychiatry Clin. Neurosci. 257, 473–479. 10.1007/s00406-007-0750-2 17902004

[B47] Schreber D. P. (2000). Memoirs of My Illness. Transl. by Macalpine I. Hunter R. A. New York, NY: New York Review Books.

[B48] Shivkumar K. Ajijola O. A. Anand I. Armour J. A. Chen P. S. Esler M.. (2016). Clinical neurocardiology defining the value of neuroscience-based cardiovascular therapeutics. J. Physiol. 594, 3911–3954. 10.1113/JP271870 27114333 PMC4945719

[B49] Sigurdsson T. Duvarci S. (2016). Hippocampal-prefrontal interactions in cognition, behavior and psychiatric disease. Front. Syst. Neurosci. 9:190. 10.3389/fnsys.2015.00190 26858612 PMC4727104

[B50] Singer T. Seymour B. O'Doherty J. Kaube H. Dolan R. J. Frith C. D. (2004). Empathy for pain involves the affective but not sensory components of pain. Science 303,1157–1162. 10.1126/science.1093535 14976305

[B51] Takahashi T. Suzuki M. Zhou S. Y. Hagino H. Tanino R. Kawasaki Y.. (2005). Volumetric MRI study of the short and long insular cortices in schizophrenia spectrum disorders. Psychiatry Res. 138, 209–220. 10.1016/j.pscychresns.2005.02.004 15854789

[B52] Vicario C. M. Nitsche M. A. Salehinejad M. A. Avanzino L. Martino G. (2020). Time processing, interoception, and insula activation: a mini-review on clinical disorders. Front. Psychol. 11:1893. 10.3389/fpsyg.2020.01893 32973605 PMC7461974

[B53] Vollmer-Larsen A. Handest P. Parnas J. (2007). Reliability of measuring anomalous experience: the Bonn scale for the assessment of basic symptoms. Psychopathology 40, 345–348. 10.1159/000106311 17657133

[B54] Wyliea K. P. Tregellas J. R. (2010). The role of the insula in schizophrenia. Schizophr. Res. 123, 93–104. 10.1016/j.schres.2010.08.027 20832997 PMC2957503

